# High‐Purity Graphitic Carbon for Energy Storage: Sustainable Electrochemical Conversion from Petroleum Coke

**DOI:** 10.1002/advs.202205269

**Published:** 2023-01-22

**Authors:** Fei Zhu, Wei‐Li Song, Jianbang Ge, Zhe Wang, Zheng Huang, Shijie Li, Mingyong Wang, Haibin Zuo, Shuqiang Jiao, Hongmin Zhu

**Affiliations:** ^1^ State Key Laboratory of Advanced Metallurgy University of Science and Technology Beijing Beijing 100083 P. R. China; ^2^ Institute of Advanced Structure Technology Beijing Institute of Technology Beijing 100081 P. R. China; ^3^ Graduate School of Engineering Tohoku University Sendai Japan

**Keywords:** energy consumption, lithium‐ion batteries, molten salt, petroleum coke

## Abstract

The petroleum coke (PC) has been widely used as raw materials for the preparation of electrodes in aluminium electrolysis and lithium‐ion batteries (LIB), during which massive CO_2_ gases are produced. To meet global CO_2_ reduction, an environmentally friendly route for utilizing PC is highly required. Here, a simple, scalable, catalyst‐free process that can directly convert high‐sulfur PC into graphitic nanomaterials under cathodic polarization in molten CaCl_2_‐LiCl at mild temperatures is proposed. The energy consumption of the proposed process is calculated to be 3 627.08 kWh t^−1^, half that of the traditional graphitization process (≈7,825.21 kWh t^−1^ graphite). When applied as a negative electrode for LIBs, the as‐converted graphite materials deliver a competitive specific capacity of ≈360 mAh g^−1^ (0.2 C) compared with commercial graphite. This approach has great potential to scale up for sustainably converting low‐value PC into high‐quality graphite for energy storage.

## Graphical abstract

The proposed molten salt electrolysis process achieves the sustainable electrochemical conversion of high‐sulfur petroleum coke into high‐purity graphitic carbon, which exhibits excellent performance as negative electrode materials of lithium‐ion batteries, and this approach paves the way for economic, sustainable, and large‐scale production of graphitic carbon materials.

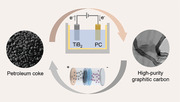

## 1 Introduction

Petroleum coke (PC), a by‐product from oil refining, is widely used in modern metallurgical industries owing to its ultra‐low cost (≈200 $ t^−1^) and abundant resource (>28 Mt a^−1^ in China).^[^ ^1^, ^2^, ^3^ ^]^The application of PC depends on the content of sulfur, a detrimental impurity that severely impedes the performance of PC. Typically, PC with low‐sulfur content (S < 0.8 wt.%), such as needle PC, is applied for the production of graphite electrodes in the steel industry, pre‐baked anodes in aluminum electrolysis, and negative electrodes in lithium‐ion batteries (LIBs).^[^ ^4^, ^5^ ^]^ The high‐sulfur (S ≥ 3 wt.%) PC, however, is directly burned as fuel (as an alternative to coal) in the cement industry and power plants, with 10% more greenhouse gas CO_2_ produced.^[^ ^6^, ^7^ ^]^

High‐sulfur PC could be converted to low‐sulfur PC by an additional desulfurization process.^[^ ^1^ ^]^ Indeed, low‐sulfur PC has been considered as an excellent raw precursor for producing high‐quality carbon due to its high carbon content and low cost. These processes in metallurgical industries, however, are complicated and energy‐intensive.^[^ ^8^ ^]^ For example, the production of graphite electrodes involves crushing, calcining, cracking, mixing, screening, shaping, repeated roasting, and energy‐intensive graphitization, giving rise to a total energy consumption of ≈7772.1 kWh t^−1^ graphite. To curb carbon emissions, on the other hand, the share of renewable energies is expected to increase in the foreseeable future. From the process design point of view, electrochemically driven techniques are expected to be of great importance to industries along with the rapid growth of renewable energies.^[^ ^9^ ^]^ Overall, it is thus urgent to explore new sustainable routes that allow the direct conversion of high‐sulfur PC to graphitic carbon with low energy consumption and reduced carbon emission. In a traditional process, the conversion of high‐sulfur PC into graphitic carbon involves a multistep process, including desulfurization, removal of other noncarbon impurities and graphitization.^[^ ^10^ ^]^ In the industries, high‐sulfur PC is roasted at ≈1500 °C to remove the sulfur attached to the carbon skeleton, during which the sulfur‐hydrocarbon compounds start to decompose and release gaseous sulfur.^[^ ^11^, ^12^ ^]^ The removal of impurities is an essential prerequisite for graphitization that is established at ultra‐high temperature ≈2900 °C.^[^ ^13^, ^14^ ^]^

Several approaches have been applied to convert PC into high‐value‐added materials, such as graphene. Saha et al. has recently demonstrated the successful conversion of PC into high‐quality graphene nanosheets using an electrochemical exfoliation (ECE) method.^[^ ^15^, ^16^, ^17^ ^]^ Mandal et al transformed PC into 2D graphitic materials by liquid exfoliation.^[^ ^18^ ^]^ Gang et al synthesized the N, Fe, and Ni co‐doped PC for the highly efficient electrochemical CO_2_ reduction reaction.^[^ ^19^ ^]^ However, carbon materials with inferior graphitization degree are usually obtained due to the low working temperature. Thus far, there is still a lack of a coupled process that enables desulfurization, impurity removal, and graphitization in a single reaction system, and more importantly, in an efficient and environmentally friendly way.^[^ ^20^, ^21^ ^]^

To substantially address the above challenges, in this work, we demonstrated a one‐step electrochemical conversion of high‐sulfur PC into graphite nanomaterials in molten CaCl_2_‐LiCl, where a constant cell voltage (2.6–3.0 V) was applied between a TiB_2_ anode and a PC pellet cathode at temperatures of 900–950 °C. This simple process directly utilizes clean electrons as the reagent for the release of impurities (S^2−^, N^3−^, O^2−^) from PC into molten salt and subsequent graphitization of PC pellet, thus avoiding desulfurization in conventional processes. In addition, the energy consumption of this molten salt approach is about half that of the typical high‐temperature treatment process, showing a promising potential to be scaled up for commercialization.

## 2 Results

### 2.1 Sustainable Production of Graphite Negative Electrodes

**Figure 1:**
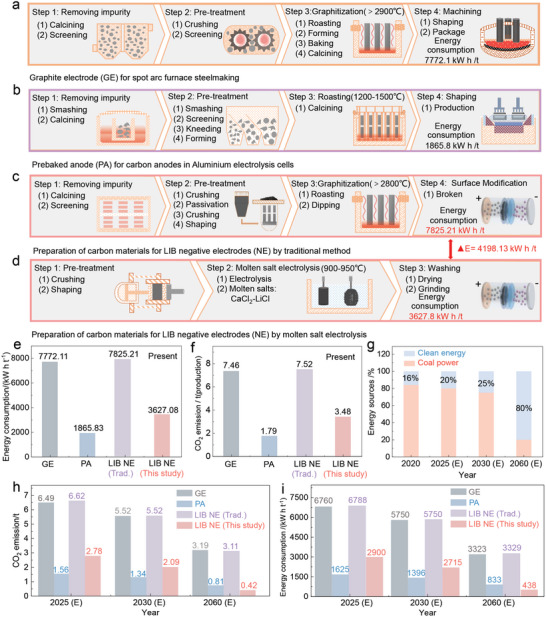
The procedures, CO_2_ emissions, and energy consumption of the processes utilizing PC as raw material for production of graphitic materials and prebaked anodes. a) Procedures of transforming PC into graphite electrode for steelmaking. b) Procedures of transforming PC into prebaked anode for aluminum electrolysis. c) Procedures of transforming PC into graphite negative electrodes for LIBs. d) Molten salt electrochemical conversion of PC into graphite negative electrodes for LIBs. e) Energy consumption of the processes. f) CO_2_ emissions of the processes. g) Future development of renewable energy in China. h) The estimated CO_2_ emission of the processes considering the developing renewable energy. i) The estimated energy consumption of the processes considering the developing renewable energy.

The utilization of PC in metallurgical industries in China is generally energy‐intensive, generating large amounts of CO_2_, as shown in **Figure** **1**. The typical process for making graphite electrodes consists of the removal of impurities (calcining and screening), the pretreatment (crushing and screening) for producing PC particles with controlled size, and the energy‐intensive graphitization (forming, baking, and calcining) in the Acheson furnace. Natural gases (275 m^3^ t^−1^ petroleum coke) are burned to produce the necessary energy for the repeated roasting; the electricity provided for the graphitization is ≈4420 kWh t^−1^ graphite, 70% of the total electricity used for the process (Note S1 and S2, Supporting Information); the energy consumption of this process is thus estimated to be 7772.1 kWh t^−1^ graphite. In the case of pre‐baked anodes (Figure 1b), the process consists of impurity removal, pretreatment, and calcining for desulfurization and strength enhancement. Despite the low graphitization of the products, the total energy consumption for the process is calculated to be 1865.8 kWh t^−1^ coke (Note S3, Supporting Information). Similar to the process of graphite electrodes, the production of negative graphite electrodes (Figure 1c) for LIB involves impurity removal, pretreatment (crushing, passivation, crushing, and shaping), energy‐intensive graphitization, and surface modification, resulting in a total energy consumption of 7825.2 kWh t^−1^ graphite (Note S4, Supporting Information). The ultra‐high temperature treatment is the primary technique to convert low‐sulfur PC to graphite electrodes and negative electrodes and consumes large amounts of energy. Compared to the current industrial processes, the proposed molten salt electrochemical approach in this study directly converts PC into graphite as a negative electrode in LIB and delivers a reduced energy consumption (Figure 1d), paving a new sustainable pathway for utilizing PC, especially the high‐sulfur PC. The total energy required for the proposed process (Figure 1d), including the pretreatment and molten salt electrolysis, is estimated to be 3627.08 kWh t^−1^ graphite (Note S5, Supporting Information), less than half that of the industrial processes. Molten salt electrolysis is at the core of the process, contributing to low energy consumption. Indeed, the theoretical cell voltage for converting high‐sulfur PC in graphite is estimated at ≈1.0 V, and to provide a sufficient electrochemical incentive to remove impurities, a cell voltage of 2.8 V is applied between a TiB_2_ anode and high‐sulfur PC cathode. Cell design with further optimization could significantly reduce the applied cell voltage. The total carbon emissions for graphite electrode, negative electrode by commercial process, negative electrode by this study, and pre‐baked anode process are calculated to be 7.46 tCO_2_/t graphite, 7.52 tCO_2_/t graphite, 3.48 tCO_2_/t graphite, and 1.79 tCO_2_/t coke, respectively, confirming the plunge in CO_2_ emission by the proposed route (Figure 1f). The use of an inert anode, TiB_2_, which generates O_2_ at the electrode surface during electrolysis, accounts for the reduced CO_2_ emissions. In addition, high‐sulfur PC, instead of low‐sulfur PC, is used as raw material for the preparation of graphite materials.

More importantly, this electrochemically‐driven technology has great potential to replace the current commercial processes along with the development of renewables in the foreseeable future. To achieve CO_2_ peak emission by 2030 and carbon neutrality by 2060, China aims to supply 20% of nonfossil energy of its energy demand by 2025; the targets set up for 2030 and 2060 are 25% and 80%, respectively (Figure 1g). Thus, the carbon emission of the proposed process is expected to decrease to 2.78 tCO_2_/t graphite in 2025, then keep declining from 2025 to 2030 (2.09 tCO_2_/t graphite), and will reach 0.42 tCO_2_/t graphite in 2060 (Figure 1h); there is, however, a limited reduction in carbon emission of the commercial processes from 2020 to 2060 owing to the use of large amounts of natural gases (Figure 1g). The energy consumption of the process supplied by power plants in 2060 could be significantly reduced to 438 kWh t^−1^ graphite as compared with that of the conventional process (3329 kWh t^−1^ graphite) (Figure 1i). Moreover, the cost of the proposed electrochemical route is calculated to be 906.5 $ per ton coke, while the value of graphite from convectional high‐temperature processes is ≈7724.7 $ per ton (Note S6, Supporting Information). Thus, high‐sulfur PC can be efficiently utilized in a green, economic way using the proposed electrochemical route.

### 2.2 Molten Salt Electrochemical Conversion of High‐Sulfur PC into Graphite

**Figure 2:**
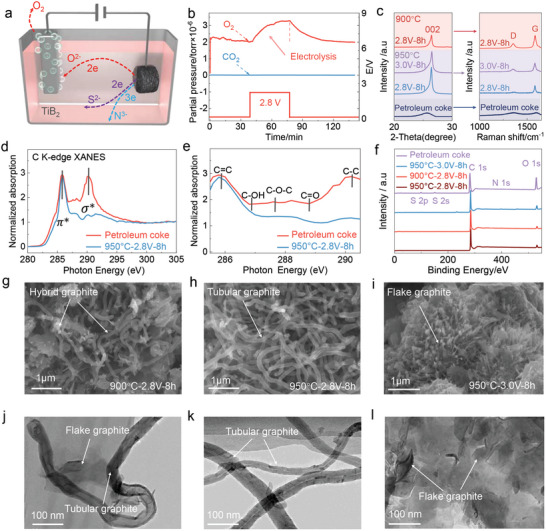
Molten salt electrochemical conversion of PC into graphitic materials. a) Schematic diagram of molten salt electrolysis. b) In situ monitoring of outlet gas during electrolysis. c) XRD and Raman results of the products obtained under the indicated electrolysis conditions. d) and e) The C K‐edge XANES spectra of PC and the product obtained after 8 h electrolysis at 950 °C and 2.8 V. f) XPS results of the products obtained under the indicated electrolysis conditions. g–i) SEM images of the hybrid graphite (g), tubular graphite (h), and flake graphite (i). j‐l) TEM images of the hybrid graphite (j), tubular graphite (k), and flake graphite (l).

We propose the electrochemical reduction of non‐carbon impurities in high‐sulfur PC as a route for its impurity removal (including desulfurization) and graphitization. A schematic diagram of the proposed electrolytic cell is shown in **Figure** **2**a. The electrolysis was conducted between a TiB_2_ anode and a high‐sulfur PC cathode in molten CaCl_2_‐LiCl.^[^ ^22^, ^23^ ^]^ Upon cathodic polarization, impurities, such as S, N, and O, were released from the high‐sulfur PC cathode into molten salt; since the dehydration of molten salt can inevitably result in the formation of little amounts of oxide (Reaction 1 and 2), we attribute the electrochemical discharge of oxygen ions (to form O_2_) to the anodic reaction (Reaction 3), which is proved by in‐situ gas chromatography (Figure 2b). The evolution of O_2_ gas confirmed the excellent stability of TiB_2_ anode during electrolysis in molten chlorides. In principle, the dissolved S^2−^ ions can also discharge at the anode surface to form elemental sulfur. The difficulty in measuring the low concentration of S^2−^ ions or collecting the trace of sulfur hinders the demonstration.

(1) $${\mathrm{CaCl}}_{2}\cdot 2H_{2}O\to \mathrm{CaO}+\mathrm{HCl}\left(g\right)$$

(2) $$\mathrm{LiCl}\cdot H_{2}O\to {\mathrm{Li}}_{2}O+\mathrm{HCl}\left(g\right)$$

(3) $$2O^{2-}-4e\to O_{2}\left(g\right)$$

The electrochemical graphitization of high‐sulfur PC under cathodic polarization was studied by X‐ray diffraction (XRD) and Raman spectroscopy (Figure 2c). The broad peak observed at low 2*θ* (20‐30°) of high‐sulfur PC indicates the characteristic of amorphous carbon. As expected, the corresponding Raman spectrum also presents the typical amorphous carbon feature with the intensity of D peak (1360 cm^−1^, disorder‐induced) comparable to that of G peak (1600 cm^−1^, sp^2^‐bond stretching vibration of a perfect graphite).^[^ ^24^, ^25^ ^]^ Generally, high‐sulfur PC was successfully converted to graphitic materials after an electrolysis time of 8 h. For example, the ratio of D peak to G peak (*I*_D_/*I*_G_, Table S1, Supporting Information) of the obtained products, is reduced to 0.14 at a cell voltage of 2.8 V and 950 °C after 8 h electrolysis. Figure 2d,e shows the corresponding normalized C K‐edge XANES spectra of the obtained product and the raw high‐sulfur PC.^[^ ^26^, ^27^, ^28^ ^]^ The absence of σ* bond demonstrates the removal of O atoms and H atoms (Note S7, Supporting Information). X‐ray photoelectron spectroscopy (XPS) analysis also confirmed the removal of non‐carbon impurities (S, N, and O), where the remaining O 1s peak results from the surface groups (Figure 2f and Figure S1, Supporting Information).^[^ ^29^, ^30^ ^]^

The scanning electron microscopy (SEM) images show three types of graphitic materials, hybrid graphite (including flake graphite and tubular graphite), tubular graphite, and flake graphite, prepared at the indicated electrolytic parameters, respectively (Figure 2g–i). The synergic effect of the insertion of the deposited lithium metal and the catalytic effect of iron mesh contributes to the formation of tubular graphite (Note S8, Figures S2–S7, Tables S2‐S3, Supporting Information). Generally, a high cell voltage (3.0 V, 950 °C) led to the agglomerated growth of tubular graphite into flake graphite, while low working temperature (2.8V, 900 °C) resulted in the formation of hybrid graphite. Moreover, the diameter of the obtained tubular graphite increased with the electrolysis time (Note S9 and Figures S5–S9, Supporting Information). Further high‐resolution transmission electron microscopy (HRTEM) reveals that hybrid graphite is comprised of flake graphite and tubular graphite (Figure 2j). The HRTEM image of tubular graphite (Figure 2k, Figure S10, Supporting Information) reveals the well‐defined crystallinity with a diameter of 46 nm, while large flake graphite can be observed in Figure 2l. Thus, by controlling the electrolytic conditions (e.g., cell voltage, electrolysis time, and working temperature), graphitic materials with various morphologies could be directly achieved.

### 2.3 Mechanism of Impurity Removal and Graphitization

**Figure 3:**
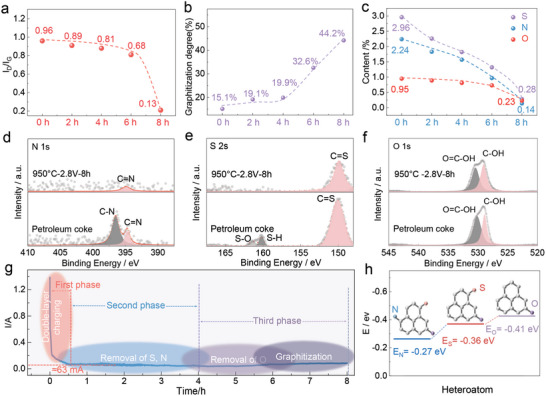
Impurity removal and graphitization mechanism of PC upon cathodic polarization. a) *I*_D_/*I*_G_ value of products at different electrolytic times under 950 °C and 2.8 V. b) The graphitization degree of products at different electrolytic times under 950 °C and 2.8 V. c) The S, N, and O content of products at different electrolytic times under 950 °C and 2.8 V. XPS results of the N 1s d), the S 2s e), and the O 1s f) for PC and the products obtained after 8 h electrolysis under 950 °C and 2.8 V. g) Typical current‐time curves obtained under 950 °C and 2.8 V. h) Removal energy of S, N, and O heteroatom from PC.

Based on the preliminary results, we identified that high‐sulfur PC could be converted into graphitic carbon, accompanied by the evolution of green O_2_ gas at the anode; however, this provides no information on how the impurity removal and graphitization proceed under cathodic polarization. The influence of electrolysis time on the graphitization process was investigated at a cell voltage of 2.8 V and 950 °C. High‐sulfur PC almost remained in its amorphous state after 2 h electrolysis (**Figure** **3**a,b). The gradual graphitization of high‐sulfur PC can be then observed with electrolysis time increasing from 2 h to 8 h (Figures S11‐S12, Supporting Information): the continuous increase in peak intensity at low 2*θ* (XRD analysis);^[^ ^31^ ^]^ the disappearance of D peak, the enhanced G peak, and the emergence of 2D peak (related to highly ordered graphite lattice). The ratio of D peak to G peak (Figure 3a, Table S1, Supporting Information), was reduced from 0.96 to 0.14. Moreover, based on the Mering–Marie equation, the calculated graphitization degree of high sulfur PC was improved from 15.1% to 44.2% after 8 h electrolysis (Figure 3b, Table S4, Supporting Information). Similar behavior can be observed at 2.8 V and 900 °C (Figures S13‐S14, Tables S5‐S6, Supporting Information).

The element analysis confirmed the gradual removal of heteroatoms from high‐sulfur PC (Figure 3c). After 4 h electrolysis, an apparent decrease of S, N, and H content could be observed. For example, the S content in the sample was decreased from ≈3.0% to 1.2%, while the H content was decreased from 3.4% to 1.8% in Table S7, Supporting Information. Intriguingly, the O content in the sample remained almost unchanged after 1 h electrolysis, and its content was only reduced from 0.9% to 0.7% after 6 h electrolysis, implying a sluggish reaction kinetics of oxygen removal. Note that the cathodic products after 6 h electrolysis still possesses a very low graphitization degree, implying the importance of oxygen removal in the graphitization process. The impurities in high‐sulfur PC maintained at a very low level after 8 h electrolysis, promoting the rearrangement of carbon atoms into the graphite lattice.

In order to further investigate the removal mechanism of heteroatoms from petroleum coke, cyclic voltammetry (CV) measurements of high‐sulfur PC and graphite (Figures S15 and S16, Supporting Information) in molten salt were performed. In the blank electrode, the CV curve yields very small currents in the range of −2.0 V–0 V (vs. Ag/AgCl), indicating the absence of electrochemical reaction; with the loading of graphite powder, the redox couple (*a*_0_/*b*_0_) appeared at −2.1 V/−0.75 V (vs. Ag/AgCl) is ascribed to the formation/reoxidation of CaC_2_. The high‐sulfur PC powder showed similar CV behavior with graphite except for the emergence of peak a_1_ at −1.1 V (vs. Ag/AgCl, Figure S15, Supporting Information). When potentiostatic electrolysis was employed and held at −1.15 V (vs. Ag/AgCl) for 5 min, the peak a_1_ disappeared, referring to the removal of the surface heteroatoms.

Based on the XPS analysis (Figure 3d‐f, Figure S1, Supporting Information), the removal of O atoms and the improved graphitization degree of PC pellet were demonstrated: the disappearance of C–O bond and the decrease in the peak intensity of sp^3^ defect carbon. The disappearance of S2p (100.2 eV), S2s (150.4 eV), and N1s (396 eV) after 8 h electrolysis illustrated the successful removal of S and N atoms during cathodic polarization. The high‐resolution spectra of S2s and N1s illustrated the elimination of S–O, S—H, and C–N bonds. The apparent decrease in peak intensity of the C=S bond (150 eV) could be also observed. Hence, we performed detailed studies to reveal the underlying mechanism. The O, S, H, and N atoms could be electrochemically released from PC cathode through the following reactions:

(4) $$O\left(C\right)+2e=\left(C\right)+O^{2-}$$

(5) $$S\left(C\right)+2e=\left(C\right)+S^{2\;-}$$

(6) $$N\left(C\right)+3e=\left(C\right)+N^{3-}$$

(7) $$2H\left(S\right)+4e=2S^{2-}+H_{2}$$

(8) $$O\left(S\right)+4e=S^{2-}+O^{2-}$$

In all, the electrochemical graphitization of high‐sulfur PC involves three steps: 1) the removal of S, N, and H; 2) the removal of O; 3) the rearrangement of disordered carbon atoms into a graphite crystal lattice. As indicated by the typical current response at 2.8 V and 950 °C (Figure 3g), the initial large current response featured the double layer charging, and rapidly decayed to ≈1 A. The current gradually decreased to 0.06 A after 4 h electrolysis, which represented the removal of S, N, and H atoms. The current remained stable in the range of 4 h–8 h, indicating the removal of oxygen and the subsequent graphitization of PC. To elucidate the impurity removal mechanism, density functional theory (DFT) calculations were utilized to determine the binding energies between conjugated carbon and heteroatoms (S, N, and O) (Figure 3h). The calculated binding energies of C–N, C–S, and C–O, were estimated to be −0.27 eV, −0.36 eV, and −0.41 eV, respectively, suggesting that the O atom is more difficult to remove than the S and N atom.^[^ ^32^ ^]^

### 2.4 Graphitic Carbon as Negative Electrodes for Lithium‐Ion Battery

**Figure 4:**
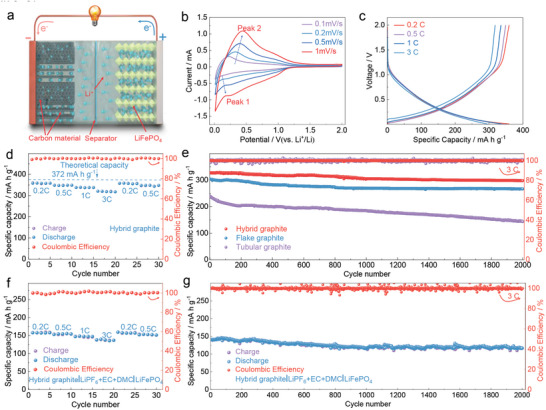
Performance of the obtained graphitic products as negative electrode for LIBs. a) Schematic diagram of LIBs. b) CV curves of hybrid graphite at various scan rates. c) The charge‐discharge curves of the hybrid graphite at various current densities. d) Rate capability of the hybrid graphite. e) Cycling performance of hybrid graphite, flake graphite, and tubular graphite at 3 C. f) Rate capability of the hybrid graphite |LiPF_6_+EC+DMC|LiFePO_4_ full battery. g) Cycling performance of the hybrid graphite |LiPF_6_+EC+DMC|LiFePO_4_ full battery at 3 C.

The prepared cathodic products (hybrid graphite, tubular graphite, and flake graphite), with high yield by SEM‐TEM, were employed as negative electrode materials for LIBs (**Figure** **4**a). (Figure 4b) shows the CV curves of the half‐cell within the voltage range of 0–2.0 V at different scanning rates.^[^ ^30^, ^33^ ^]^ Correspondingly, the hybrid graphite as a negative electrode for LIBs possesses a high specific surface area of 627.28 m^2^ g^−1^ (Figure S17, Supporting Information). In the CV curves of half‐cell in Figure S18a, Supporting Information, the area of the first circle is obviously larger than that of the subsequent circles, suggesting that a large amount of lithium metal is consumed to form a solid electrolyte interface film in the first cycle. A pair of distinct redox peaks were observed in the CV curves, which refers to the intercalation/dissolution of Li^+^ ions into/out of graphitic materials. The charging and discharging specific capacities of hybrid graphite are 311.4 mA h g^−1^ and 425.3 mA h g^−1^ at 3C rate in Figure S18b, Supporting Information. Although the large surface area of hybrid graphite consumes a large amount of lithium, resulting in a low initial Coulombic Efficiency, hybrid graphite can exhibit outstanding rate performance, ascribed to the highly efficient intercalation/dissolution process of Li‐ions. The galvanostatic charge/discharge test was carried out to evaluate the energy storage behavior of the prepared carbon nanomaterials. (Figure 4d) depicts the rate performance of the prepared hybrid graphite at different current densities of 0.2–3 C. The discharge capacity of the half‐cell remains very steady at 358.7, 348.9, 337.8, and 320.5 mA h g^−1^ at 0.2, 0.5, 1, and 3 C, respectively, demonstrating a favorable rate capability. When the current density returns to 0.2 and 0.5 C, the specific capacities reach 357.1 and 348.2 mA h g^−1^, comparable to the theoretical capacity of commercial graphite. The corresponding charge and discharge curves are shown in (Figure 4c). Moreover, these carbon nanomaterials all exhibit good cycling stability beyond 2000 cycles at a current density of 3C (Figure 4e), of which the hybrid graphite presents the highest specific capacity of about 320.1 mA h g^−1^ with a coulombic efficiency of almost 100%.

To further evaluate the performance of hybrid graphite, a full battery was assembled with a LiFePO_4_ positive electrode, which delivers stable reversible capacities of 157.9, 153.6, 147.3, and 137.4 mA h g^−1^ at the current densities of 0.2, 0.5, 1, and 3 C, respectively (Figure 4f). When the current density returns to 0.2 and 0.5 C, the specific capacity could increase to 157.6 mA h g^−1^ and 153.1 mA h g^−1^, respectively, indicating its excellent rate capability. After 2000 cycles, the cell could still deliver a reversible capacity of 132.3 mA h g^−1^ with coulombic efficiency close to 100% (Figure 4g), implying a stable and reversible electrochemical reaction in this full battery upon charge/discharge tests.

## 3 Discussion

### 3.1 Carbon‐Reduction Incentives to Sustainable Utilization of PC

The present industrial processes of utilizing PC could generate large amounts of CO_2_, especially in the case of high‐sulfur PC, which is directly burned as fuel in cement plants and power plants. In aluminum industry, at least 0.42 tons of prebaked anode (derived from low‐sulfur PC) must be used for every ton of aluminum produced (Reaction 9), not mention to the enormous energy consumption for aluminum production (≈13,500 kWh t^−1^ Al).^[^ ^34^ ^]^ In addition, graphite electrodes are also applied as reductants for the production of silicon, as shown in (Reaction 10). However, to alleviate the increase in its energy consumption and accomplish the carbon peak emission goal, China requires the development of new zero‐carbon emission technologies and the increase in the share of renewable energy sources.^[^ ^35^, ^36^ ^]^ For example, a higher percentage of aluminum metal would be supplied from the aluminum recycling process in the foreseeable future. In such a scenario, the demand for low‐sulfur PC is expected to decline, which leads to an imbalance in PC cycles. With the increasing share of renewable energies, high‐quality graphite is highly required in the near future due to its wide application in energy storage systems. Indeed, low‐sulfur PC is applied as a main raw precursor for preparing graphite electrodes and negative electrodes. However, the typical conventional processes consume large amounts of energy due to the energy‐intensive graphitization process. It is thus urgent to develop new sustainable routes, especially methods driven by renewables, that can convert PC into graphite materials with low energy consumption and carbon emissions.

(9) $$C+{\mathrm{Al}}_{2}O_{3}=\mathrm{Al}+{\mathrm{CO}}_{2}$$

(10) $$C+{\mathrm{SiO}}_{2}=\mathrm{Si}+{\mathrm{CO}}_{2}$$

### 3.2 Features of the Molten Salt Electrochemical Approach

In industries for producing graphitic carbon, the processes mainly involve the repeated roasting for desulfurization and energy‐intensive graphitization. Typically, PC is roasted at temperatures of 1300–1600 °C to remove the attached sulfur. Several other approaches were also considered, such as solvent extraction, oxidative desulfurization, sulfur‐bearing gas treatment, hydrocarbon gas treatment, and alkali metal treatment. Despite their high efficiency, these methods involve the use of reactants, which increase the cost, and still require a high temperature to accelerate the reaction rate. The typical ultra‐high temperature treatment is made in an Acheson furnace at ≈2900 °C, consuming 70% of the total electricity. The routes towards graphitization with low energy consumption have long been pursued. The catalytic graphitization of PC with Fe_2_O_3_ at 800–900 °C, but the removal of this catalyst remains a major concern. Recently, Peng et al verified the direct transformation of amorphous carbon into graphite nanoflakes through cathodic polarization in molten salt.^[^ ^24^ ^]^ Nevertheless, there is still a lack of a coupled process that enables desulfurization and graphitization to occur in a single reaction system.

On the basis of the analysis, the developed approach in the present study has several advantages. First, molten salt electrolysis is conducted in an electrolytic cell, where desulfurization, impurity removal, and graphitization of high‐sulfur PC could be all accomplished at a relatively low temperature of 950 °C. This simplicity not only greatly lowers the total energy requirement but also reduces the capital cost for industry‐scale synthesis. Second, the molten salt electrolytes used in this study are cost‐effective, easy to operate, and possess a wide electrochemical window, which allows the graphite synthesis to occur at a considerable current density. Lastly, molten salt can be easily removed by water after electrolysis, without any contamination of the produced carbon materials. This is of great importance to produce high‐purity graphite materials.

The proposed process has great potential to be scaled up for graphite synthesis owing to the principle of molten salt electrolysis. Aluminum electrolysis is currently operated at a scale of 600 kA for the production of primary aluminum, which would serve as a guide for the commercialization of the proposed process. The findings of this study show superior advantages over conventional processes, while other factors, such as the morphology and quality control at the industry‐scale, are needed to be investigated for ongoing research.

## 4 Conclusion

In summary, instead of applying ultra‐high temperature treatment, we prove that graphitization can proceed by the reduction of non‐carbon impurities to form soluble ions (such as O^2−^, S^2−^, and N^3−^) in a molten salt media at relatively low temperatures of 900–950 °C. The process is simply conducted in a single molten salt reactor, and is, therefore, more sustainable. More importantly, the prepared hybrid graphite could deliver a capacity of 320.5 mAh g^−1^ at 3 C after 2000 cycles, comparable to that of commercial graphite. This approach paves the way for economic, sustainable, and large‐scale production of negative electrode materials for lithium‐ion battery.

## 5 Experimental Section

### Materials

Anhydrous CaCl_2_, LiCl, and AgCl of analytical purity were purchased from Aladdin reagent Co., Ltd. Iron mesh, tungsten, and silver wire (purity > 99.9%) were provided by Kang Wei Co., Ltd. Graphite rod was purchased from Jun Rong Co., Ltd. TiB_2_ was provided by Jia Ming Co., Ltd. Glassy carbon rod was purchased from Shanghai Xian Ren Co. Ltd. Boron nitride was obtained from Kai Fa Co., Ltd. N‐methyl‐2‐pyrrolidone (NMP) solvent, LiPF_6_ and LiFePO_4_ were purchased from Mackcin Co., Ltd. Lithium metal, Cu foil, and Al foil were provided by BaiHua Co., Ltd. Acetylene black and polyvinylidene fluoride (PVDF) was purchased from QiYue Co. Ltd. High sulfur petroleum coke (PC) was provided by Qi Lu Petrochemistry Co., Ltd.

### Molten Salt Electrochemical Conversion of PC

An alumina crucible containing 100 g CaCl_2_ with 10 wt.% LiCl was put into a vertical tubular furnace. Then the salts were dried at 300 °C for 10 h under vacuum to remove moisture and subsequently heated up to the desired temperatures (900 or 950 °C) under argon atmosphere. After the operating temperature was reached, the system was left to stabilize for 1 h. To remove impurities and residual water in molten salt, pre‐electrolysis was conducted with a W working electrode and a graphite rod counter electrode for 8 h. Then electrolysis and electrochemical testing were performed in this system (Figure S19, Supporting Information). The PC powder was ground into smaller particles (<75 µm) and then dried at 120 °C for 10 h. A 0.6 g of the prepared PC powder was pressed into pellets with a diameter of 15 mm and thickness of 2 mm, using a uniaxial pressure of 2 MPa. Then the pellet was wrapped by an iron mesh to form the cathode in constant cell voltage electrolysis. TiB_2_ and Ag/AgCl electrodes served as anode and reference electrode, respectively. Constant cell voltage electrolysis was performed between a PC pellet cathode and a TiB_2_ anode via an electrochemical workstation (AMETEK, PARSTAT 4000A). The electrolysis was performed at 2.6 V, 2.8 V, and 3.0 V with different electrolysis times of 1 h, 2 h, 4 h, 6 h, 8 h, and 10 h. After the electrolysis, the electrodes were raised near the top of the reactor and left to cool. The cathodic products were taken out and rinsed with 1 M HCl solution and deionized water to remove the frozen electrolyte, and then dried in a drying oven at 150 °C. Cyclic voltammetry (CV) test was carried out using a fabricated boron nitride indentation electrode (BNIE). The BNIE was made using a boron nitride tube (2 mm in inner diameter, 6 mm in outer diameter, and 50 mm in length) and a glass carbon rod (2 mm in diameter and ≈58 mm in length) in Figure S20, Supporting Information. The left edge of the BNIE electrode showed a cylindrical groove where the PC powder was compacted into the interior of the boron nitride tube (depth 2 mm). The glass carbon rod was connected to the PC powder serving as a current collector. The other edge of the BNIE, the glass carbon rod, was physically inserted into a graphite rod (6 mm diameter, 150 mm length). CV measurements were conducted at 100 mV s^−1^ using Solartron S1 1287 workstation (UK). The BNIE loaded with PC powders or electrolytic products and a 6 mm graphite rod were used as the working electrode and the counter electrode, respectively. The Ag/AgCl electrode was selected as the reference electrode. The I_D_/I_G_ values of graphitized carbon materials were fitted with Origin software, and the ratio of half height to width was used to calculate the graphitization degree in Figure S21, Supporting Information. Finally, the online mass spectrometer was used to detect the gas of TiB_2_ anode. Considering the low oxygen content in the petroleum coke and the large environment in the furnace chamber, a new anode cover was designed to accurately measure in Figure S22, Supporting Information.

### Electrochemical Measurements of the Electrolytic Products as LIBs Negative Electrode

The electrochemical performance of three electrolytic products (hybrid graphite, tubular graphite and flake graphite) was evaluated as negative electrodes for Li‐ion batteries using the CR2032 coin cell. The electrolytic products were mixed with acetylene black and polyvinylidene fluoride (PVDF) with a mass ratio of 7:2:1 and dispersed in N‐methyl‐2‐pyrrolidone (NMP) solvent. The formed slurry was coated on Cu foil and dried at 60 °C for 10 h in vacuum oven, and was further cut into an electrode with a diameter of 12 mm. The mass loading of the electrolytic products in the electrode was ≈0.6 mg. The LiFePO_4_ electrode was made using the above method with Al foil as a current collector. The electrolytic products electrodes were assembled with Li metal and LiFePO_4_ electrode into a half cell or a full cell for testing, respectively. The CV was carried out with a CHI 660E electrochemical workstation. The galvanostatic charge/discharge of all batteries were performed with a Neware battery testing system. All the batteries were assembled in an argon glove box ([O_2_] < 0.1 ppm, [H_2_O] < 0.1 ppm).

### Characterization

The pristine PC and all electrolytic products were characterized by X‐ray diffraction analysis (XRD, Rigaku, D/max‐RB), Raman spectrometer (Horiba‐Labram HR evolution, an excitation at 532 nm), scanning electron microscopy (SEM, JEOL, JSM‐6701F), transmission electron microscopy (TEM, JEOL 2100F), and X‐ray photoelectron spectroscopy (XPS, Kratos, AXIS Ultra DLD). C K‐edge spectra of pristine PC and electrolytic product (950 °C–2.8V–8h) were obtained under ambient conditions at 4B9A beamline of the Beijing Synchrotron Radiation Facility, and all X‐ray absorption spectroscopy (XAS) spectra were normalized using Athena software.

## Conflict of Interest

The authors declare no conflict of interest.

## Supporting information

Supporting Information

## Data Availability

The data that support the findings of this study are available from the corresponding author upon reasonable request.
