# Supplementary material for: High‐Purity Graphitic Carbon for Energy Storage: Sustainable Electrochemical Conversion from Petroleum Coke
Source: Adv Sci (Weinh). 2023 Jan 22;10(8):2205269. doi: 10.1002/advs.202205269 (PMC10015905; doi:10.1002/advs.202205269)
Supplement: Supplementary file 1 — Supporting Information [file ADVS-10-2205269-s001.pdf]

ADVANCED SCIENCE
Open Access

Supporting Information

for *Adv. Sci.*, DOI 10.1002/advs.202205269

High-Purity Graphitic Carbon for Energy Storage: Sustainable Electrochemical Conversion from Petroleum Coke

Fei Zhu, Wei-Li Song, Jianbang Ge, Zhe Wang, Zheng Huang, Shijie Li, Mingyong Wang, Haibin Zuo, Shuqiang Jiao* and Hongmin Zhu*

Supporting Information

High-purity graphitic carbon for energy storage: Sustainable electrochemical conversion from petroleum coke

Fei Zhu, Wei-Li Song, Jianbang Ge, Zhe Wang, Zheng Huang, Shijie Li, Mingyong Wang, Haibin Zuo, Shuqiang Jiao*, and Hongmin Zhu*

F. Zhu, Prof. J. Ge, Prof. Z. Wang, Prof. M. Wang, Prof. H. Zuo, Prof. S. Jiao, Prof. H. Zhu.
State Key Laboratory of Advanced Metallurgy
University of Science and Technology Beijing
Beijing, 100083, P R China
E-mail: mywang@ustb.edu.cn (M. Wang), sjiao@ustb.edu.cn
Prof. W-L Song, S. Li, Prof. S. Jiao
Institute of Advanced Structure Technology
Beijing Institute of Technology
Beijing 100081, P R China
Prof. H. Zhu
Graduate School of Engineering,
Tohoku University, Sendai, Japan.
F. Zhu, W. -L. Song and J. Ge contributed equally to this work

Supplementary Note 1: Calculation of energy consumption

The petroleum coke is used to produce graphite electrodes, graphite negative electrodes, and prebaked anodes. The energy consumption mainly comes from coal power and natural gas. The energy consumption of the processes was calculated and the specific formula is as follows[1-4] :

The specific heat capacity of carbon, Cp (J/K) :

$$Cp=1.8586+1.2893\times10^{-4}T-2.5647\times10^{5}T^{-2}+4.5197\times10^{5}T^{-3} \quad (S1)$$

where T is the applied temperature (K).

The theoretical heat consumption of carbon or graphite products is introduced from:

$$\int_0^T Cp\ dT \quad (S2)$$

Combining formula (S1) with (S2):

$$H_L=1.8586T+6.4465\times10^{-5}T+2.5649\times10^{5}T^{-1}-2.5599\times10^{7}T^{-2} \quad (S3)$$

H_L: Theoretical energy consumption for heating carbon or graphite, kJ/kg.

The natural gas, V_L(m^3/t), needed to provide the necessary thermal energy is:

$$V_L=0.02813\times H_L \quad (S4)$$

The electricity needed to provide the thermal energy can be calculated:

$$W_L=0.2778\times H_L \quad (S5)$$

W_L: The consumed electricity for providing the necessary thermal energy, kWh/t.

As is well known, 1 kWh of the electricity from power plants can release 0.96 kg CO_2. Burning 1 m^3 natural gas can provide 9.884 kWh.

The actual energy consumption is calculated according to the heat utilization rate of different heating furnaces.

Supplementary Note 2: Energy consumption for producing graphite electrodes

Graphitization accounts for 70% of the electricity and the heat utilization efficiency of the Acheson furnace is 49%, and the graphitization temperature is 2900 °C, while the roasting temperature is set to 1500 ℃.

T_1=3173 K, T_2=300 K, it follows:

H_{T1}=5897.33+649.03+80.83-2.25=6624.94 kJ/kg

H_{T2}=557.58+5.8+854.97-251.9=1166.45 kJ/kg

$\triangle H_{\text{Graphite elecrrodes}} = H_{T1} - H_{T2}$ =5458.49 kJ/kg

Actual energy consumption:

$W_{\text{Graphite elecrrodes}} = 0.2778 \times \triangle H_{\text{Graphite elecrrodes}} \div 49\% \div 70\%$ =4420 kWh/t

The estimated energy consumption of calcination for the pre-removal of heteroatoms is about 634 kWh/t. The total energy consumption from the electricity is: 5054 kWh/t.

Natural gas is used in the following processes: primary roasting, impregnation and secondary roasting.

The natural gas consumption is:

$V_{\text{Graphite elecrrodes}}$=275 m^3/t

The energy from natural gas is:

275m^3/t×9.884 (kWh/m^3) =2718.1 kWh/t

The total energy consumption is: 5054+2718.1= 7772.1 (kWh/t)

Therefore, 4.85 t CO_2 from power plants and 2.61 t CO2 from the burning of natural gas are released, respectively, for every ton of graphite.

Supplementary Note 3: Energy consumption for production prebaked anode

The prebaked anode process refers to the thermal treatment of PC at 1300 ℃.

T_1=1573 K, T_2=300 K, it follows:

H_{T1} = 3237.03 kJ/kg

H_{T2} = 1166.45 kJ/kg

$\Delta H_{prebaked\ anode} = H_{T1} - H_{T2} = 2070.58$ kJ/kg

The energy consumption from electricity is:

$W_{prebaked\ anode} = 0.2778 \times \Delta H_{prebaked\ anode} \div 49\% = 1173.89$ kWh/t

Natural gas is used in the following: roasting and impregnation.

The natural gas consumption:

$V_{negative\ electrodes} = 70\ m^3/t$

The energy consumption from natural gas:

$70\ m^3/t \times 9.884(kWh/m^3) = 691.88$ kWh/t

The total energy consumption is: 1173.89+691.88= 1865.8 (kWh/t)

Therefore, 1.13 t CO_2 from power plants and 0.66 t CO_2 from the burning of natural gas are released, respectively, for every ton of pre-baked anodes.

Supplementary Note 4: Energy consumption for producing negative electrodes

The process of graphite negative electrode and graphite electrode is similar, but graphite negative electrode has passivation, surface modification and other processes than graphite electrode process. Graphitization accounts for 70% of coal power and the heat utilization rate of the series graphitization furnace is 49%, the graphitization temperature is calculated at 2800 ℃.

T_1=3073 K, T_2=300 K, as follows:

H_{T1}=6401.31 kJ/kg

H_{T2}=1166.45 kJ/kg

$\Delta H_{negative\ electrodes} = H_{T1} - H_{T2} = 5234.86$ kJ/kg

Actual energy consumption:

$W_{negative\ electrodes} = 0.2778 \times \Delta H_{negative\ electrodes} \div 49\% \div 70\% = 4239.47$ kWh/t

Natural gas is used in the following: roasting and impregnation.

The estimated energy consumption of calcination pre-removal of heteroatoms, passivation and surface modification is about 976.4 kWh/t.

The natural gas consumption:

$V_{\text{negative electrodes}}$=264 m^3/t

The energy consumption from natural gas:

264m^3/t×9.884(kWh/m^3)=2609.34 kWh/t

The total energy consumption is: 5215.87+2609.34= 7825.21 (kWh/t)

Therefore, 5.01 t CO_2 from power plants and 2.51 t CO_2 from the burning of natural gas are released, respectively, for every ton of negative graphite electrodes.

Supplementary Note 5: Energy consumption for producing carbon nanomaterials by molten salt electrolysis

Different carbon materials are obtained by treating petroleum coke. The molten salt electrolysis temperature is in the range of 900-950 ℃. In the one-step molten salt electrochemical conversion process, the energy consumption comes from the heating process and the electrolysis process[5]. During the heating process, petroleum coke and molten salt was heated from 25 ℃ to 950 ℃. When the cell voltage is 2.8 V, the charge flowing through the system is 1890 C. The calculated details are shown as follows:

$M_{\text{petroleum coke}}$:12g/mol;

$M_{\text{molten salt}}$:111g/mol;

1Cal=4.184J;

$\triangle_{\text{petroleum coke}}$ and $\triangle_{\text{molten salt}}$: The energy needed for heating PC and molten salt from 298 K to 1223 K, respectively.

(1) The energy consumption during electrolysis:

$W_{\text{electrolysis}}$ =1890 (C)×2.8 (V)÷3600 (s) = 1470 kWh/t

(2) Cathode (Petroleum coke):

$\Delta H_{\text{Petroleum coke}}$ =4.008(T_1=1223 K)-0(T_2=298 K) = 4.008 kJ/mol

Energy: 4.008×1000÷12=334 kJ

Energy consumption: 334×10^3×4.1814÷3600 = 387.9 kWh/t

(3) The evaporated molten salt during electrolysis:

$\Delta H_{\text{Molten salt}}$ =-161.58(T_1=1223 K) +190(T_2=298 K) = 28.42 kJ/mol

Energy: 28.42×1000÷111=256 kJ

Energy consumption: 256×10^3×4.1814÷3600 =318.6 kWh/t

The total energy consumption is: (1470+387.9+318.6)/60% = 3627.5 (kWh/t)

Therefore, ~3.48 t CO_2 is released for every ton of carbon nanomaterials prepared by molten salts.

Supplementary Note 6: Cost estimation

In order to reflect the cost of molten salt electrochemical conversion of petroleum coke into high value carbon material, we made a preliminary cost estimate. First, the raw material used in the traditional process of high temperature graphitization is low sulfur PC, while high sulfur petroleum coke is directly used as cathode in the present process. The market price of high sulfur petroleum coke ranges from 125.1 $/ton to 202.3 $/ton, and that for low sulfur petroleum coke is in the range of 576.9 $/ton to 734.5 $/ton. Second, for the cost of energy consumption, using an electricity cost of 0.1 $/kWh,[6] the total energy consumption of molten salt electrolysis process is estimated to be 3627.8 kWh/ton. Thus, the energy cost for the proposed process is 362.8 $/ton coke. Third, the labor cost and capital expense can be calculated based on the industrial molten salt electrolysis. Taking aluminum electrolysis as an example, the corresponding labor cost and capital expense are 150 $/ton, respectively. The cost of anode preparation and molten salt are considered to be negligible since they are not

consumed during electrolysis. Considering the evaporation of molten $CaCl_2$-LiCl (30 kg/ton coke), the added cost is roughly estimated to be 80 \$/ton coke. Therefore, the total cost for the electrochemical conversion of high-sulfur coke into graphite is calculated to be 906.5 \$/ton coke (assume that the cost of high sulfur PC is 163.7 \$/ton). Typically, the market prices of graphite electrode and graphite negative electrode from the conventional high-temperature graphitization process, are 10252.8 \$/ton and 7724.7 \$/ton, respectively.[7,8] Thus, the electrochemical conversion of high-sulfur PC into graphite materials has a strong economic incentive.

Supplementary Note 7: C K-edge XANES spectra of the petroleum coke

C K-edge XANES spectra of the petroleum coke and petroleum coke-after (950°C, 2.8V, 8h) was conducted. Based on the dipole-transition selection rules, the C K-edge STXM-XANES can be attributed to electron transitions from the C 1s core-level to the 2p final unoccupied states of petroleum coke and petroleum coke-after. The magnified π* region clearly shows the excited state of C= C at 285.8 eV and the wide features of (C-OH)- (C-C) in the range 286.8-290.2 eV between the features π* and σ* of C-C, corresponding to specific oxygen-containing and hydroxyl groups in both flat and wrinkle regions of petroleum coke and petroleum coke-after. Although the assignment of these features to specific chemical states has been controversial, these features are typically attributed to the chemical states of the C 2p that is bound to oxygen and hydrogen atoms, respectively, specific to π* (C-OH) at ~286.8 eV, to (C-O-C) at ~287.6 eV, to C=O in the -COOH bond at ~288.8 eV and to C=O at ~290.2 eV. The lower energy of the p*-feature in the petroleum coke spectrum relative to that of the petroleum coke-after can be attributed either to the high degree of structural rearrangement of the aromatic ring that is decorated with the oxygen-containing functional groups or to the effect of edge/defect states.

Supplementary Note 8: Formation mechanism of tubular graphite

The formation of tubular graphite could be related to the insertion of liquid Li nanoparticles. The decomposition cell voltage for molten LiCl at 950 °C was calculated to be 3.2 V (**Reaction S6**). Thus, the applied cell voltage (2.6 V, 2.8 V and 3. 0 V) was not be able to electrochemically decompose LiCl to produce liquid Li metal and Cl_2 gas. However, small amounts of O^{2-} ions was generated during the vacuuming process for the removal of moisture in $CaCl_2$ (**Reaction S7** and **S8**). Then the decomposition of Li_2O to form Li and O_2 gas could take place at a much lower cell voltage (~2.2V at 950 °C, **Reaction S9**). The other explanation for the mechanism is the catalytic effect of Fe mesh. It has been reported that Fe could serve as nucleation cite for the electrochemical growth of carbon nanomaterials in molten carbonates.[9,10] It should be pointed out there is no evidence for the formation of lithium metal or lithium carbide, which could be eliminated in the rinsing process for the removal of frozen electrolyte.

$$2LiCl = Li + Cl_2 \quad (S6)$$

$$CaCl_2{\cdot}H_2O = CaO + 2HCl \quad (S7)$$

$$CaO + 2LiCl = CaCl_2 + Li_2O \quad (S8)$$

$$2Li_2O = 4Li + O_2 \quad (S9)$$

To investigate the effects of Li^+ ions and Fe mesh on the formation of tubular graphite, constant cell voltage electrolysis under various conditions (**Table S3**) was conducted at 2.8 V and 950 °C for 8 h. As can be seen in **Figure S2a** and **b**, no tubular graphite were observed in pure $CaCl_2$ with or without the application of iron mesh. The XRD and Raman results in **Figure S3** and **S4** illustrated the products were comprised of amorphous carbon, which implied an incomplete removal of heteroatoms from PC pellet after electrolysis in pure $CaCl_2$. Note that the application of iron mesh slightly increased the graphitization degree (from 18.9 to 20.1%) of PC after electrolysis. The results proved that iron mech had almost no influence on the removal of heteroatoms from PC pellet. When the LiCl was added into $CaCl_2$, small amounts of tubular graphite were formed (**Figure S2c**). More intriguingly, the addition of

LiCl greatly enhanced the graphitization process. As can be seen in **Table S2** and **Table S3**, the graphitization degree of PC (without the application of iron net) after the electrolysis was increased to 36.1% and its I_D/I_G ratio was calculated to be 0.51. Thus, the formed liquid Li nanoparticles not only resulted in the formation of small amounts of tubular graphite, but also promoted the removal of heteroatoms and accelerated the graphitization process. The application of iron mesh in molten $CaCl_2$-LiCl produced a considerable amount of tubular graphite (**Figure S9e** and **Figure S5**), implying the excellent catalytic effect of Fe on the formation tubular graphite. To demonstrate this, 10 wt% Fe powder was added into PC pellet for constant cell voltage electrolysis. As shown in **Figure S2d**, massive tubular graphite could be observed after 8 h electrolysis. The initial multimodal size distribution (**Figure S5** and **S6**) turned into a monomodal size distribution (**Figure S7**) after the addition of Fe powder. The diameter of the obtained tubular graphite ranged from ~8 nm to 72 nm with a median diameter centered at ~26 nm. Furthermore, the added Fe powder resulted in the increasing amount of tubular graphite as compared to the products in **Figure S7**. the introduction of LiCl into molten salt led to the deposition of Li metal during electrolysis. It is well known that [O] in TiO_2 could be removed by Ca metal in molten $CaCl_2$ in OS process.[11,12] Additional chemical reduction of [O] or [S] in PC pellet by Li metal accelerated the removal of heteroatoms (**Reaction S10**, **S11** and **S12**). After the removal of heteroatoms, the reorganization of carbon atoms proceeded and tubular graphite formed under the iron mesh catalysis.

$$O(C) + 2Li = (C) + O^{2-} + 2Li^{+} \quad \text{(S10)}$$

$$S(C) + 2Li = (C) + S^{2-} + 2Li^{+} \quad \text{(S11)}$$

$$N(C) + 3Li = (C) + N^{3-} + 3Li^{+} \quad \text{(S12)}$$

Supplementary Note 9: Morphology evolution

The morphology changes before and after electrolysis under 950 °C were characterized by scanning electron microscopy (SEM). As shown in (**Figure S8** and **S9**), the original PC

was mainly comprised of irregular lumps. The lump of carbon material in micro-size could also be observed in the products obtained under 2.6 V and 8 h electrolysis. At 2.8 V and 950 ℃, the products after 1, 2 and 4 h electrolysis exhibited irregular micro-lump morphologies. The SEM image after 4 h of electrolysis showed the emergence of very tiny tubular graphite (dia. ~5.8 nm). When the electrolysis time was prolonged to 6 h and 8 h, the formation and growth of large amounts of tubular graphite could be observed. Diameter distributions of tubular graphite were taken from image analysis using multiple SEM images from multiple spots on the products.

Based on the outer diameter, the tubular graphite grown from 6 h electrolysis exhibited a multimodal size distribution from ~8 to 84 nm (**Figure S5**). The corresponding peaks were centered at ~22 nm, 24 nm, 38 nm and 76 nm, respectively. The distribution was mainly comprised of small tubular graphite with a diameter ranging from 12 nm to 38 nm. Similar size distribution could be observed for the tubular graphite obtained from 8 h electrolysis (**Figure S6**). The corresponding peaks were centered at ~22 nm, 27 nm, 63 nm and 74 nm, respectively. And the results indicated a larger quantity of tubular graphite after 8 h electrolysis compared to the products after 6 h electrolysis.

Figure S1. XPS results of C 1s spectra for pristine PC and the product obtained after 8 h electrolysis under 2.8 V and 950 ℃.

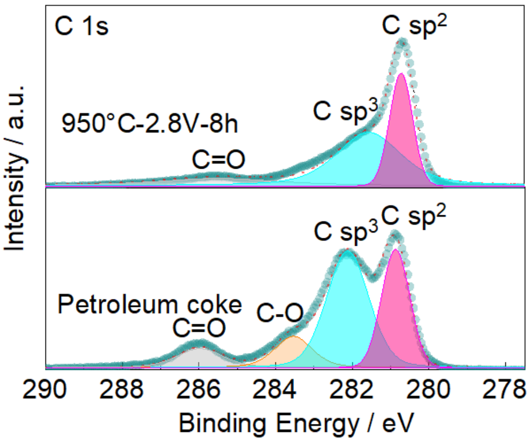

XPS was further employed to confirm the electrochemical graphitization process and the removal of heteroatoms from PC after 8 h of electrolysis under 2.8 V and 950 ℃. There are two distinct peaks at around 281.5 eV and 531.5 eV, corresponding to the C1s and O1s. The high-resolution spectra of C1s peak could be divided into four peaks: sp^2 graphite carbon (281.6 eV), sp^3 defect carbon (282.3 eV), C-O bond (284.1 eV) and C=O bond (285.9 eV).

Figure S2. SEM images of PC after electrolysis (950 ℃, 2.8 V and 8 h) under different conditions: a) In $CaCl_2$ without the application of Fe net; b) In $CaCl_2$ with the application of Fe net; c) In $CaCl_2$-LiCl without the application of Fe net; d) In $CaCl_2$-LiCl with the application of Fe net+10% Fe added into PC pellet

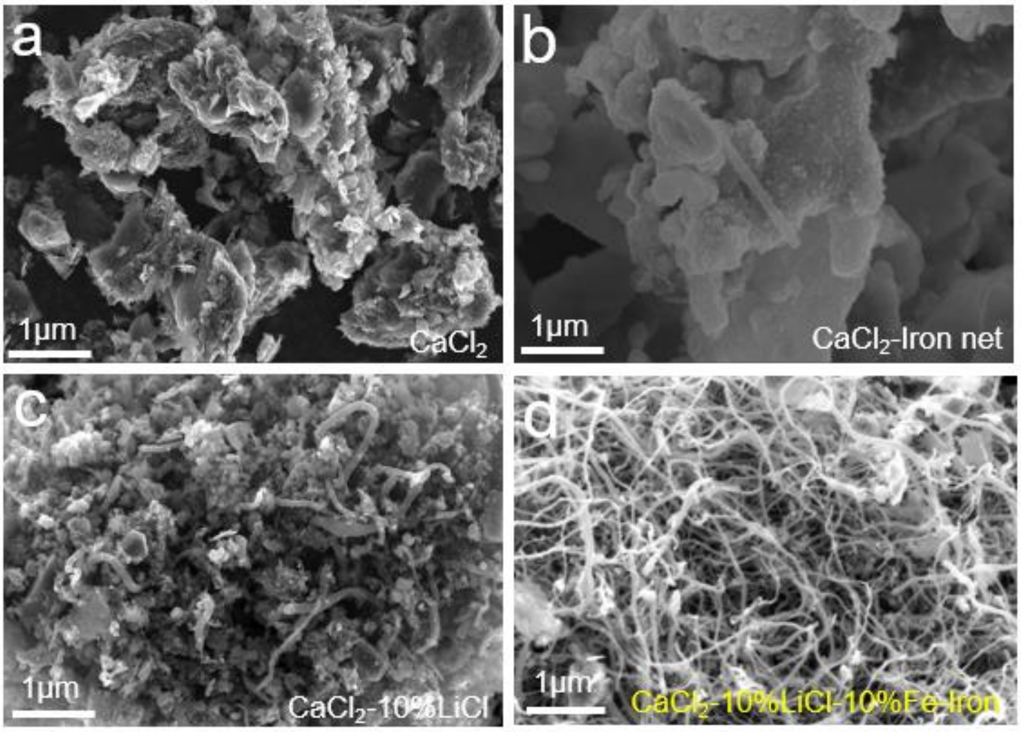

Figure S3. XRD patterns of PC after electrolysis under different molten salt system

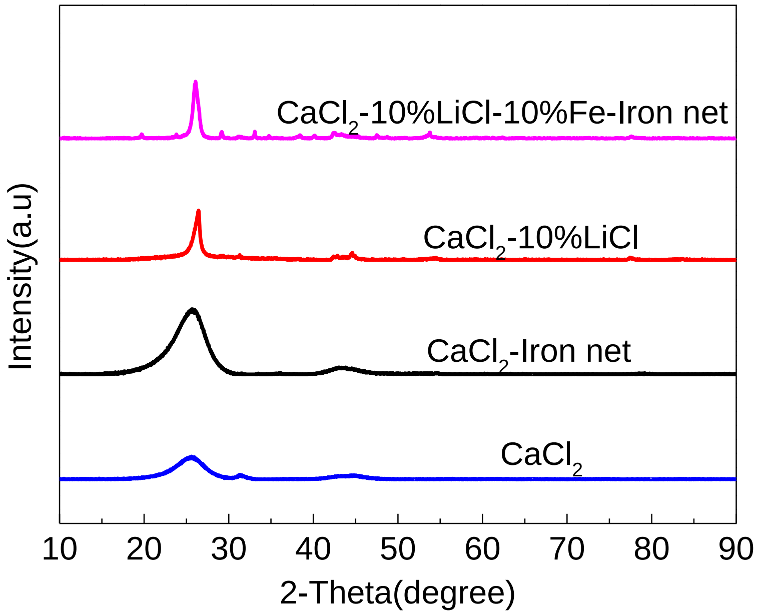

Figure S4. Raman patterns of PC after electrolysis under different molten salt system

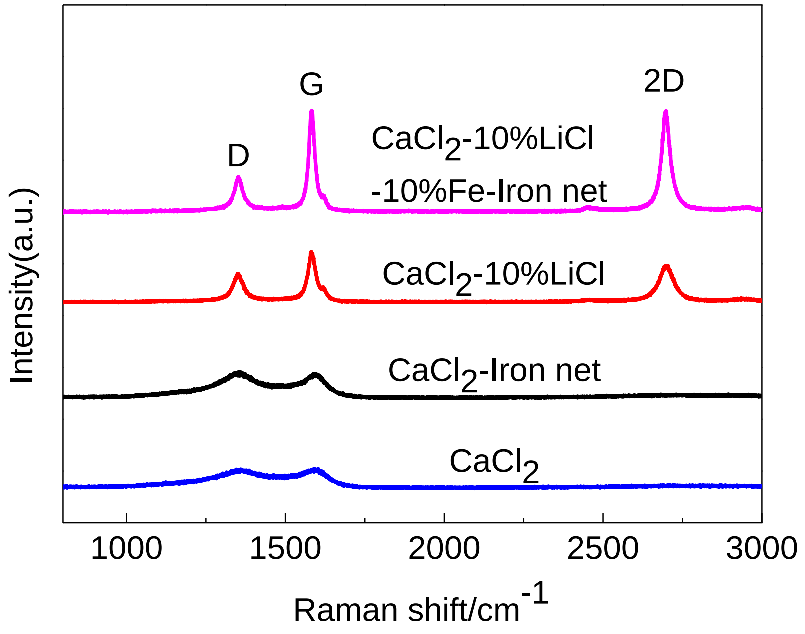

Figure S5. SEM images and size distributions of the tubular graphite obtained under 950 ℃ and 2.8 V for 6 h electrolysis. a-c) The SEM images of the tubular graphite. d)The size distributions of the tubular graphite.

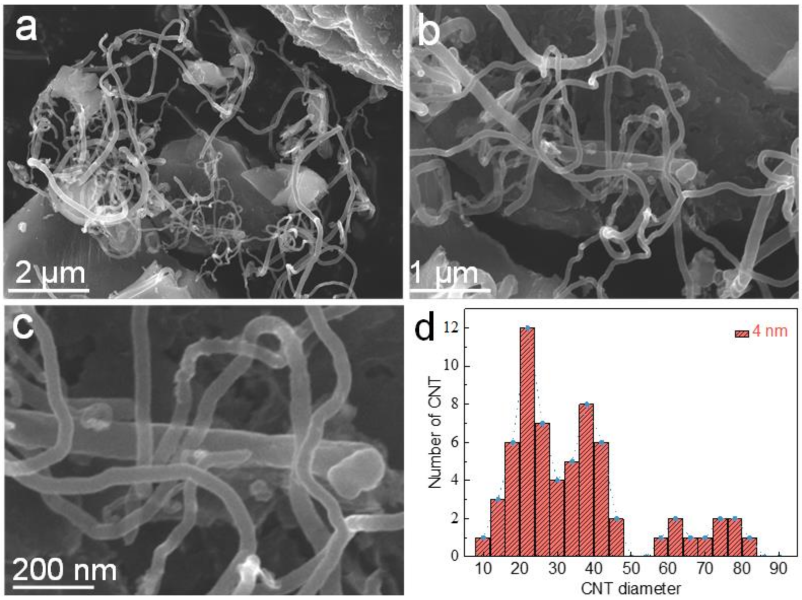

Figure S6. SEM images and size distributions of tubular graphite obtained under 950 ℃ and 2.8 V for 8 h electrolysis. a-c) The SEM images of the tubular graphite. d) The size distributions of the tubular graphite.

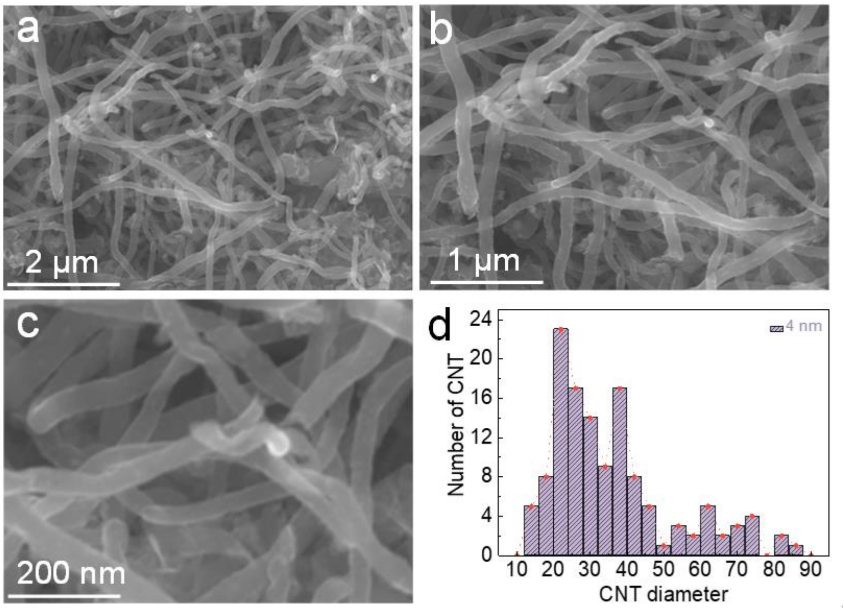

Figure S7. Size distributions of the obtained tubular graphite in PC-10wt%Fe pellet after 8 h electrolysis under 950 ℃ and 2.8V.

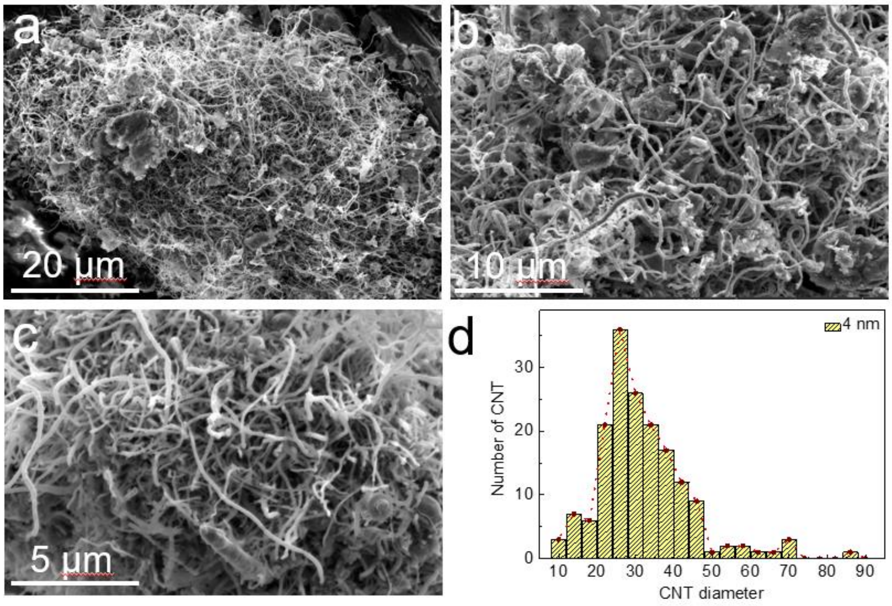

Figure S8. SEM images of the products obtained under the indicated electrolytic conditions at 900 ℃.

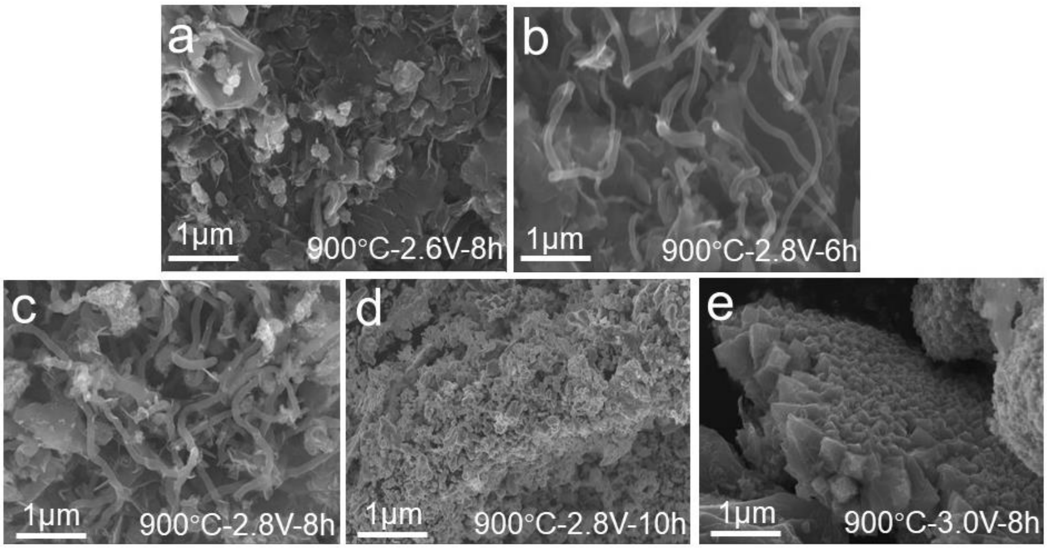

Figure S9. SEM images of pristine PC and the products obtained under various electrolytic conditions at 950 ℃. a) The SEM image of pristine PC, b-i) The SEM images of product obtained under the indicated electrolysis conditions.

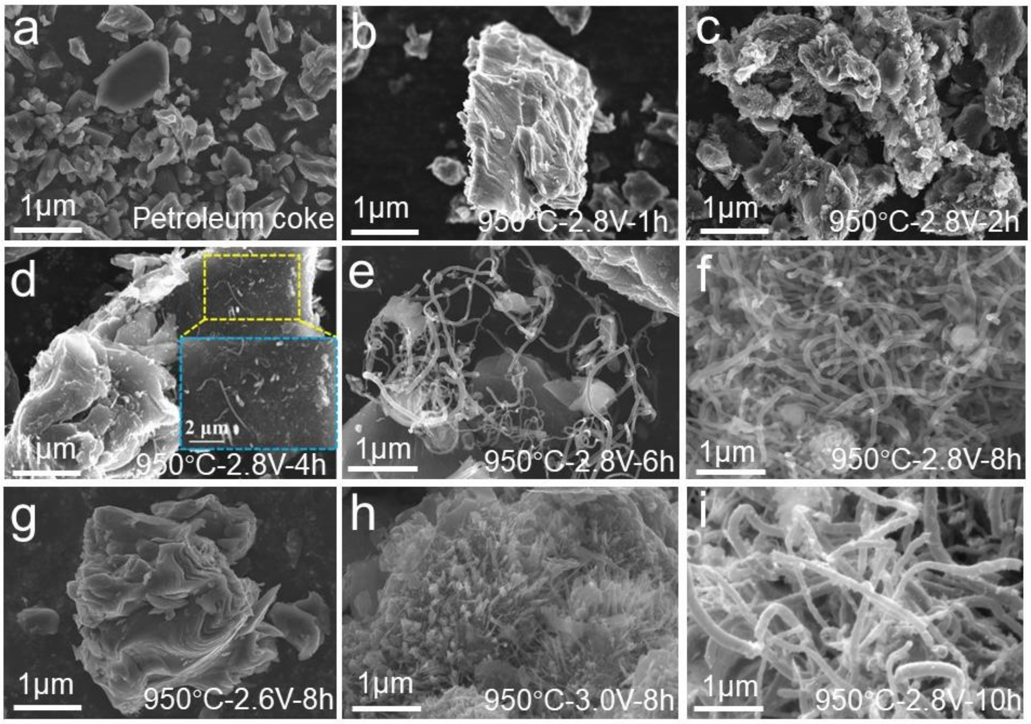

Figure S10. TEM images of hybrid graphite obtained under 950 °C and 2.8 V for 8 h electrolysis. The hybrid graphite is composed of flake graphite and tubular graphite.

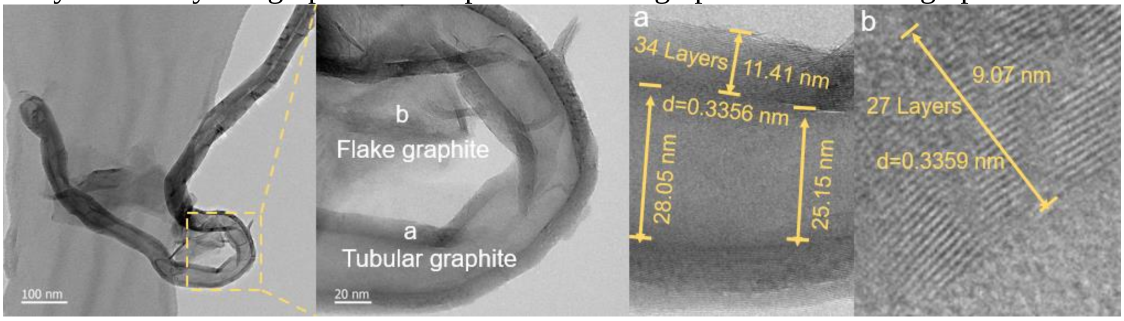

Figure S11. XRD patterns of pristine PC and the products obtained under various electrolytic conditions at 950 ℃. The product obtained after 8 h electrolysis at 2.6 V and 950 ℃ is shown '950 ℃-2.6V-8h' in the figure.

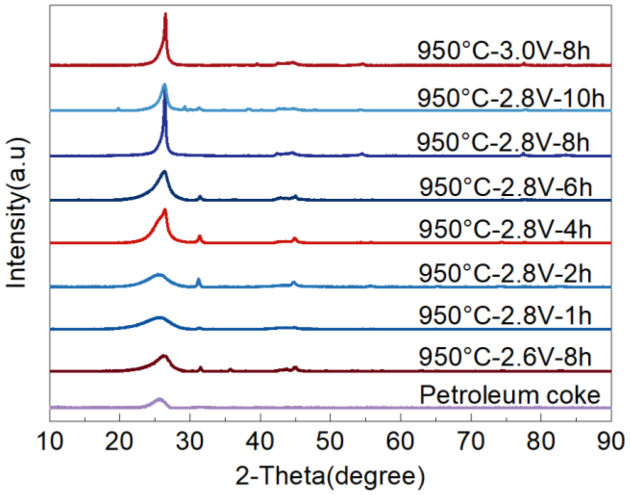

Figure S12. Raman patterns of pristine PC and the products obtained under various electrolytic conditions at 950 ℃.

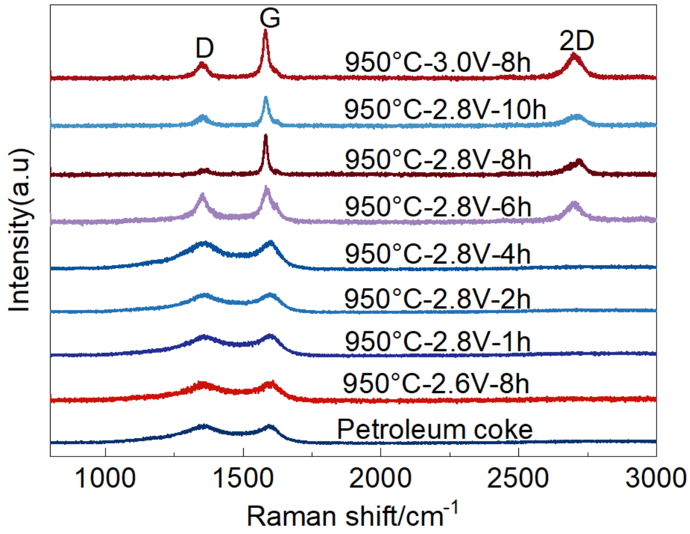

Figure S13. XRD patterns of the products obtained under various electrolytic conditions at 900 °C.

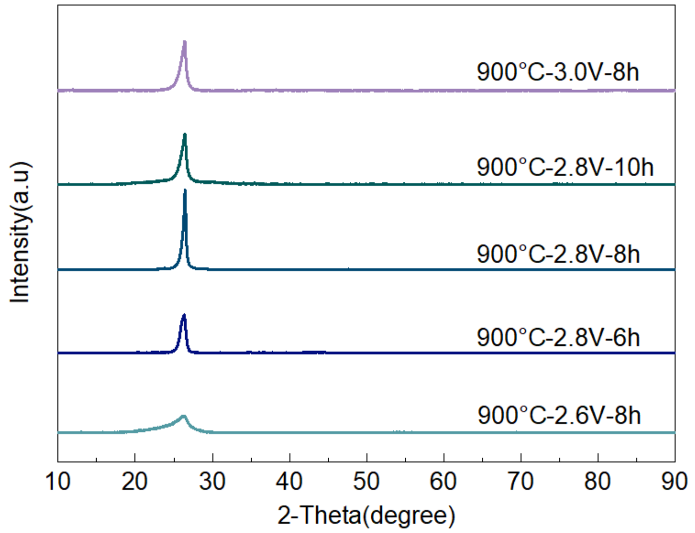

Figure S14. Raman patterns of the products obtained under various electrolytic conditions at 900 °C.

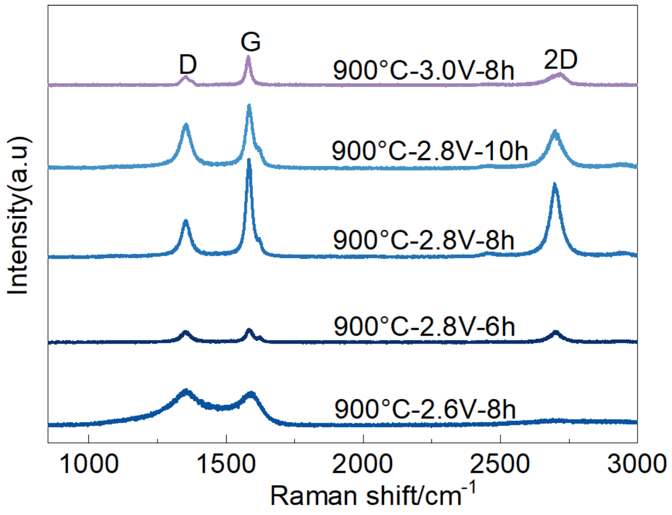

Figure S15. CV curves of BNIE and petroleum loaded BNIE (BNIE+ petroleum) in $CaCl_2$-10wt%LiCl melts at a scan rate of 100 mV/s.

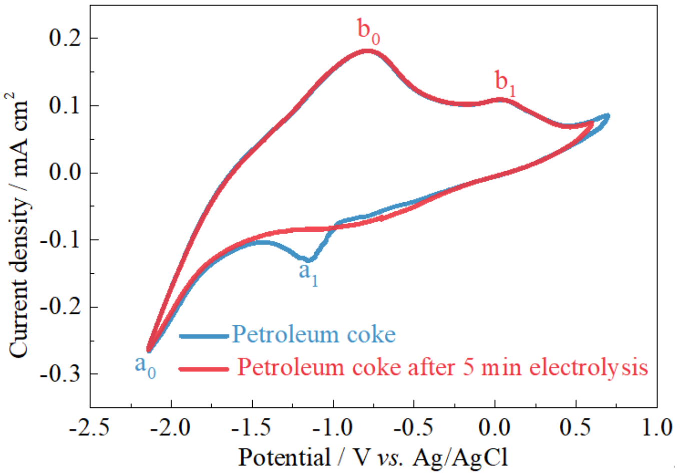

Figure S16. CV curves of BNIE and graphite loaded BNIE (BNIE+Graphite) in $CaCl_2$-10wt%LiCl melts at a scan rate of 100 mV/s.

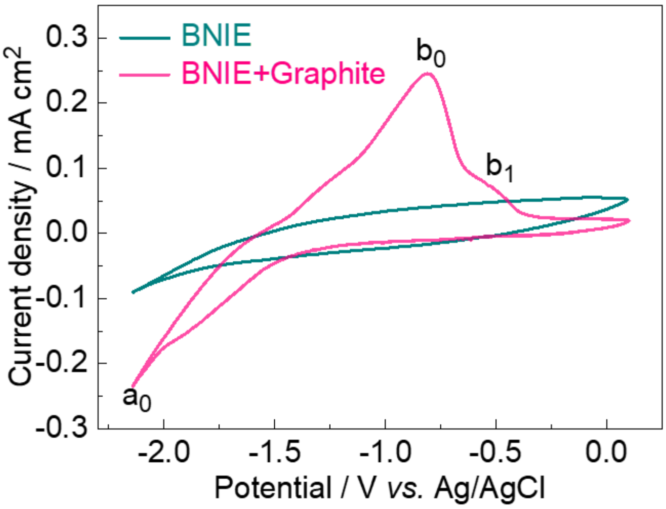

Figure S17. The specific surface area of petroleum coke and hybrid graphite

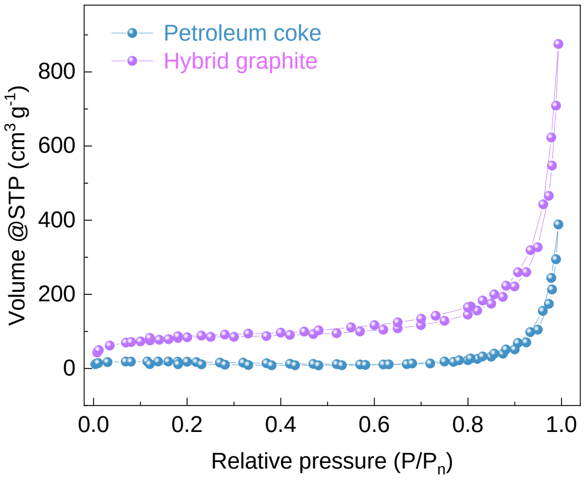

Figure S18. The initial two cycles in CV curves a) and initial charge-discharge curves b) of Li-based half cells assembled with hybrid graphite.

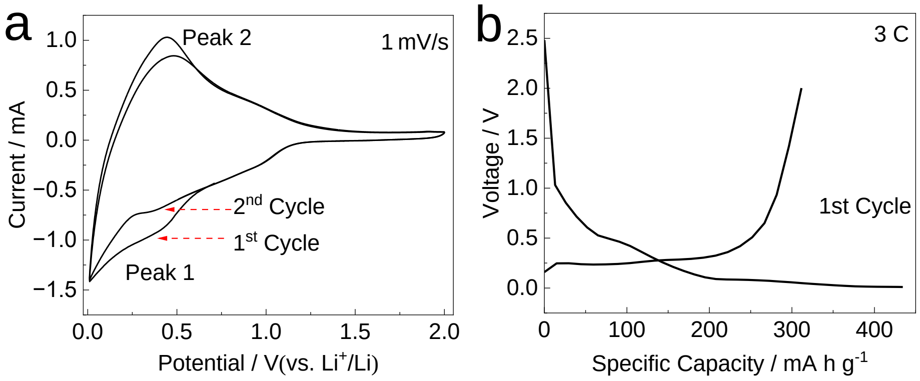

Figure S19. The schematic diagram of molten salt electrochemical conversion of PC. The anode is TiB_2 which is enclosed in a glass cover that collects gases during the electrolysis process. The PC powder is pressed and packed with iron mesh, and serves as cathode during electrolysis in molten $CaCl_2$-LiCl.

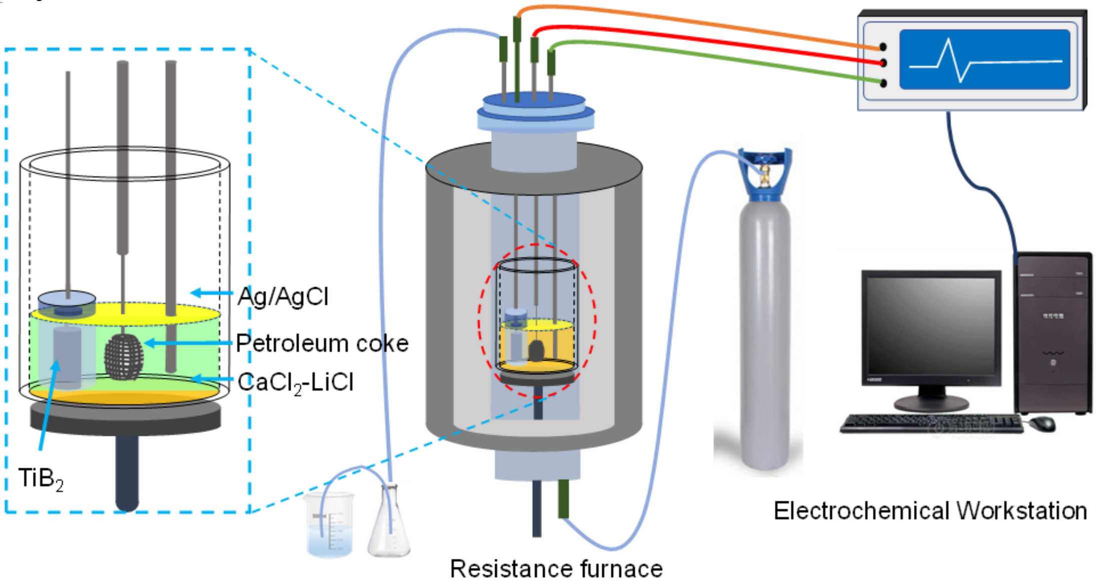

Figure S20. The digital photo and schematic diagram of boron nitride indentation electrode (BNIE). The BNIE consists of a carbon rod and a boron nitride tube with a cavity and the sample is placed into the cavity during testing.

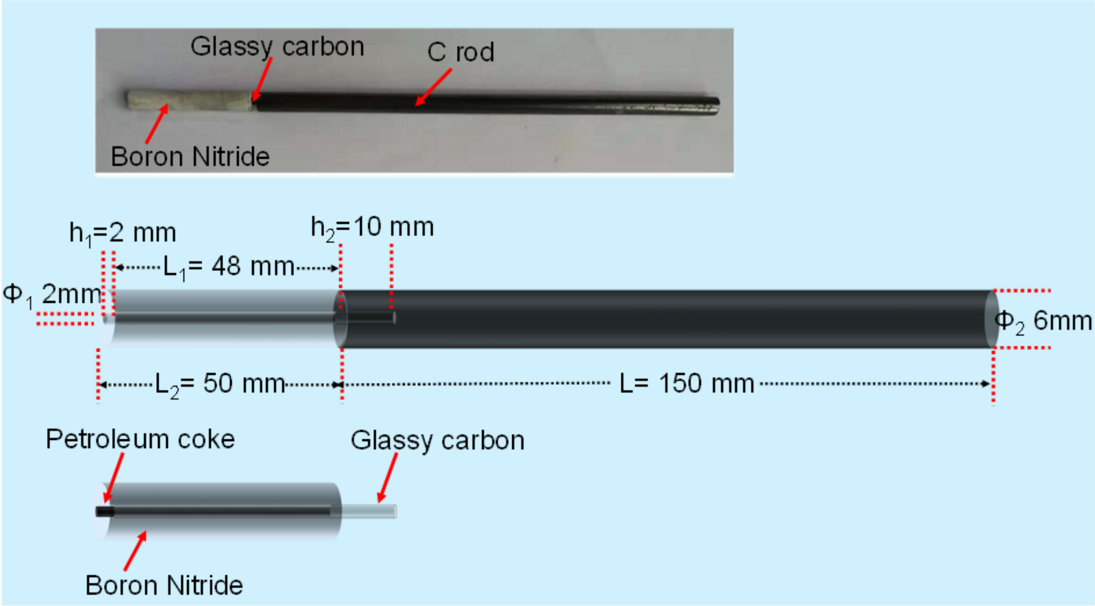

Figure S21. Calculation of I_D/I_G values of graphitized carbon materials at 900 °C, 2.8 V, 8 h

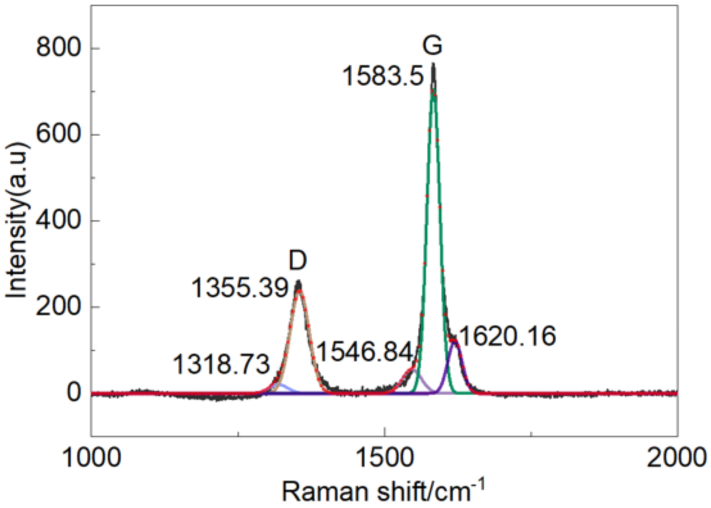

Taking the carbon material obtained at 900 °C and 2.8 V after 8 h electrolysis as an example, the I_D/I_G of the sample is obtained by the ratio of D peak to the half-height width of peak G (I_D/I_G =237.15/709.01=0.33). In the meantime, the five peaks of region corresponding are 1318.73 cm^{-1}, 1355.39 cm^{-1}, 1546. 84 cm^{-1}, 1583.5 cm^{-1}, and 1620.16 cm^{-1} respectively.

Figure S22. The photograph of TiB_2 anode and PC cathode. The TiB_2 is 4 cm long and is covered by glass with an open end. The PC powder is pressed into block with a 15mm diameter and packed with iron mesh.

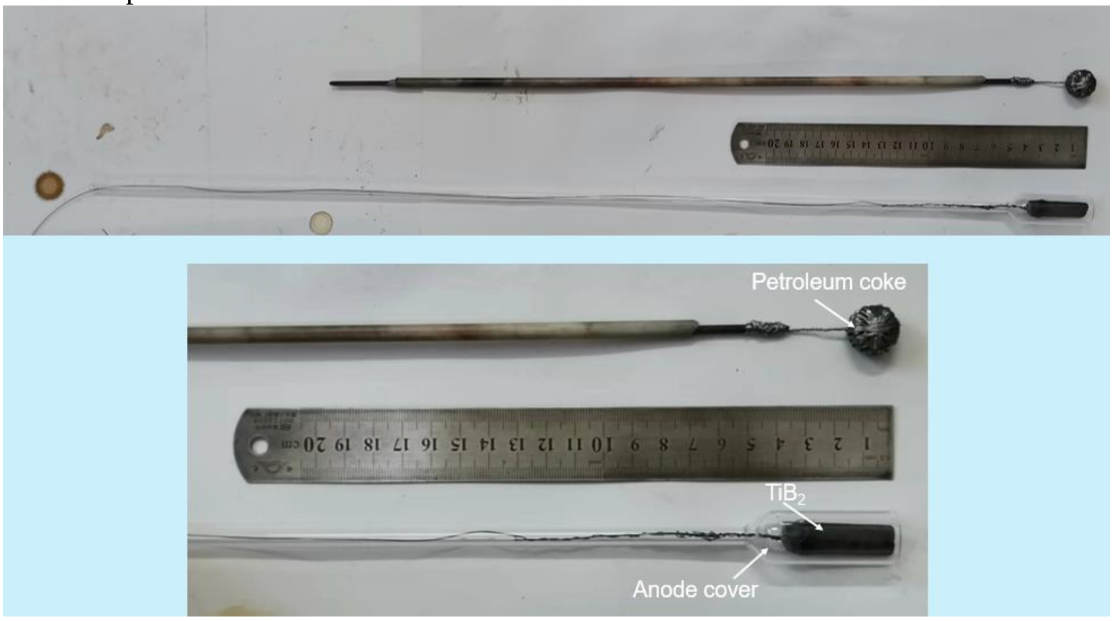

Table S1. The I_D/I_G values of pristine PC and the products obtained under various electrolytic conditions at 950 °C.

Condition	D peak Position (cm^{-1})	G peak Position (cm^{-1})	I_D/I_G
950℃-3.0V-8h	1337.14	1579.67	0.3933
950℃-2.8V-10h	1336.15	1582.26	0.3537
950℃-2.8V-8h	1353.14	1585.68	0.135
950℃-2.8V-6h	1343.14	1579.67	0.681
950℃-2.8V-4h	1342.46	1578.24	0.8024
950℃-2.8V-2h	1340.38	1571.39	0.8875
950℃-2.8V-1h	1336.17	1575.76	0.9031
950℃-2.6V-8h	1331.12	1573.68	0.9243
Petroleum coke	1320.14	1572.68	0.9598

Table S2. The calculated graphitization degree of PC after electrolysis (950 ℃, 2.8 V and 8 h) under different molten salt system.

Molten salt system	θ	g/%
$CaCl_2$	13.006	18.92
$CaCl_2$-Iron Net	13.028	20.04
$CaCl_2$-10%LiCl	13.053	36.01
$CaCl_2$-10%LiCl-10%Fe-Iron Net	13.064	38.75

Table S3. The I_D/I_G values of PC after electrolysis (950 ℃, 2.8 V and 8 h) under different molten salt system.

Molten salt system	D peak Position (cm^{-1})	G peak Position (cm^{-1})	I_D/I_G
$CaCl_2$	1320.75	1571.93	0.9473
$CaCl_2$-Iron Net	1328.26	1572.08	0.9316
$CaCl_2$-10%LiCl	1339.41	1579.32	0.5128
$CaCl_2$-10%LiCl-10%Fe-Iron Net	1335.17	1581.25	0.3127

Table S4. The calculated graphitization degree of pristine PC and the products obtained under various electrolytic conditions at 950 ℃.

Condition	θ	g/%
950℃-3.0V-8h	13.069	39.53
950℃-2.8V-10h	13.072	40.16
950℃-2.8V-8h	13.081	44.19
950℃-2.8V-6h	13.045	32.56
950℃-2.8V-4h	13.017	19.87
950℃-2.8V-2h	13.009	19.13
950℃-2.8V-1h	13.002	18.65
950℃-2.6V-8h	13.058	36.74
Petroleum coke	12.993	15.12

Table S5. The I_D/I_G values of the products obtained under various electrolytic conditions at 900 ℃.

Condition	D peak Position (cm^{-1})	G peak Position (cm^{-1})	I_D/I_G
900℃-3.0V-8h	1342.14	1575.6	0.4633
900℃-2.8V-10h	1349.14	1579.68	0.4378
900℃-2.8V-8h	1345.14	1580.63	0.3314
900℃-2.8V-6h	1343.14	1575.68	0.649
900℃-2.6V-8h	1340.13	1578.68	0.9257

Table S6. The calculated graphitization degree of PC and the products obtained under various electrolytic conditions at 900 ℃.

Condition	θ	g/%
900℃-3.0V-8h	13.056	36.05
900℃-2.8V-10h	13.062	38.37
900℃-2.8V-8h	13.072	43.75
900℃-2.8V-6h	13.049	34.88
900℃-2.6V-8h	13.021	25.58

Table S7. The elemental contents in pristine PC and the products obtained under various electrolytic conditions at 950 ℃.

Sample	C	N	S	O	H
950℃-3.0V-8h	93.52	0.39	0.31	0.79	1.93
950℃-2.8V-10h	95.37	0.18	0.26	0.21	0.76
950℃-2.8V-8h	95.69	0.14	0.28	0.23	0.81
950℃-2.8V-6h	94.34	0.97	1.32	0.73	1.86
950℃-2.8V-4h	91.26	1.57	1.82	0.81	2.48
950℃-2.8V-2h	89.34	1.83	2.26	0.89	2.69
950℃-2.8V-1h	85.15	2.06	2.58	0.93	3.09
950℃-2.6V-8h	89.65	1.98	2.29	0.91	2.68
Petroleum coke	84.72	2.24	2.96	0.95	3.41

References

[1] I. J. Okeke, T. A. Adams, *Can. J. Chem. Eng.* **2021**, *99*, S340.

[2] H. Mousavi, J. Khodadadi, *Physica E.* **2013**, *50*, 11.

[3] M. G. Xia, Y. Song, S. L. Zhang, *Phys. Lett. A.* **2011**, *375*, 3726.

[4] M. Johnson, J. Ren, M. Lefler, G. Licht, J. Vicini, S. Licht, *Data Brief.* **2017**, *14*, 592.

[5] W. Weng, L. Z. Tang, W. Xiao, *J. Energy Chem.* **2019**, *28*, 128.

[6] J. Lau, G. Dey, S. Licht, *Energ. Convers. Manage*. **2016**, *122*, 400.

[7] M. Johnson, J. W. Ren, M. Lefler, G. Licht, X. Y. Liu, *Mater. Today Energy.* **2017**, *5*, 230.

[8] J. M. Beer, *Prog. Energy Combust. Sci.* **2007**, *33*, 107.

[9] A. Douglas, R. Carter, M. Y. Li, C. L. Pint, *ACS Appl. Mater. Interfaces.* **2018**, *11*, 19010.

[10] X. R. Wang, F. Sharif, X. Y. Liu, G. Licht, S. Licht, J. *CO_2 Util.* **2020**, *40*, 101218.

[11] R. O. Suzuki, K. Teranuma, K. Ono, *Metall. Mater. Trans. B.* **2003**, *34*, 287.

[12] R. O. Suzuki, *J. Phys. Chem. Solids.* **2005**, *66*, 461.
